# First person – Amy Findlay

**DOI:** 10.1242/dmm.038265

**Published:** 2018-12-18

## Abstract

First Person is a series of interviews with the first authors of a selection of papers published in Disease Models & Mechanisms, helping early-career researchers promote themselves alongside their papers. Amy Findlay is first author on ‘Mouse *Idh3a* mutations cause retinal degeneration and reduced mitochondrial function’, published in DMM. Amy is a postdoc in the lab of Ian Jackson at MRC Human Genetics Unit, University of Edinburgh, Edinburgh, UK. The focus of her research is using mouse models of human disease to investigate the genetic causes of retinal degeneration.


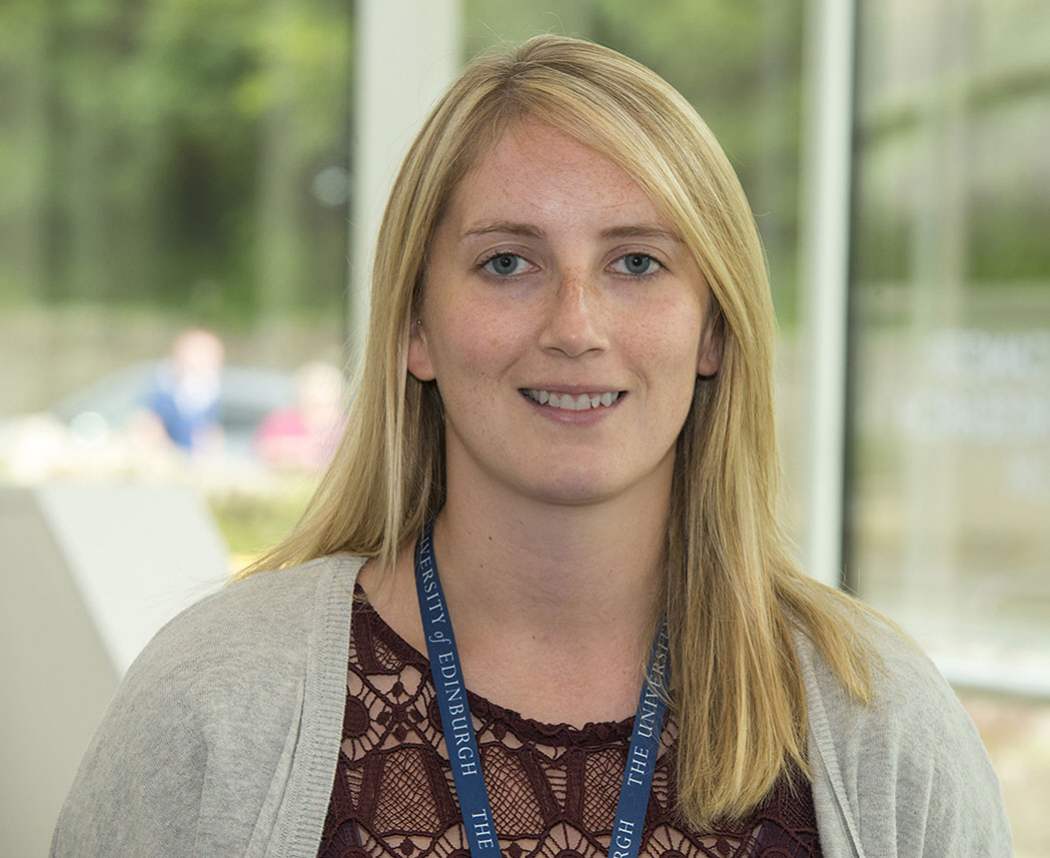


**Amy Findlay**

**How would you explain the main findings of your paper to non-scientific family and friends?**

We are interested in how the genes inherited from your parents can affect eyesight and cause blindness. We have looked at how a mutation – or change – in one gene, *Idh3a*, causes childhood blindness. This gene is important in the function of the mitochondria – the part of the cell that produces energy – and, when mutated, mitochondria cannot produce as much energy. This causes blindness, as the light-sensitive cells in the retina, the photoreceptors, need a lot of energy to function. If the mitochondria are not producing the high levels of energy these cell need, they cannot function properly and die.

“We have shown that a mitochondrial defect present in all cells has a detrimental effect only on photoreceptor cells.”

**What are the potential implications of these results for your field of research?**

By understanding the mechanism behind photoreceptor loss, we are better equipped to develop therapeutics for prevention or treatment in patients. We have shown that a mitochondrial defect present in all cells has a detrimental effect only on photoreceptor cells.

**What are the main advantages and drawbacks of the model system you have used as it relates to the disease you are investigating?**

Mice are a great model of human disease and, as shown in our paper, the *Idh3a* mutants exhibit early and rapid degeneration just like the human patients. However, *Idh3b* mutants do not seem to replicate the late-onset human disease, suggesting that the mouse model may be different from humans. In addition, complete loss of IDH3A results in early embryonic death, whilst loss of IDH3B has little or no effect on development and physiology, demonstrating the different functions of the two enzyme subunits.

**What has surprised you the most while conducting your research?**

The largest surprise is that the mitochondrial phenotype is present in other cells, not just the photoreceptors, but it appears as though only the photoreceptors are affected by the reduced function.

**Describe what you think is the most significant challenge impacting your research at this time and how will this be addressed over the next 10 years?**

As genetic testing becomes more common, the volume of genetic information of patients is increasing at an exponential rate. The greatest challenge facing my research is the ability to analyse all the patient data and assess how the many variants cause the phenotypes observed. This better understanding is required for the diagnosis and treatment of patients.

“[…] the ‘publish or perish’ climate that many early-career scientists are forced into is detrimental to both the scientist and also science as a whole.”

**What changes do you think could improve the professional lives of early-career scientists?**

I echo the concerns of a large portion of the interviewed authors and say that the ‘publish or perish’ climate that many early-career scientists are forced into is detrimental to both the scientist and also science as a whole. In a time when we should be developing our skills required for our future careers, we are urged to think short-term. This can either result in talent leaving the field or, in some cases, unethical practices. Something that could alleviate this stress is higher-impact journals recognizing that negative results are important and worth reporting.

**Figure:**
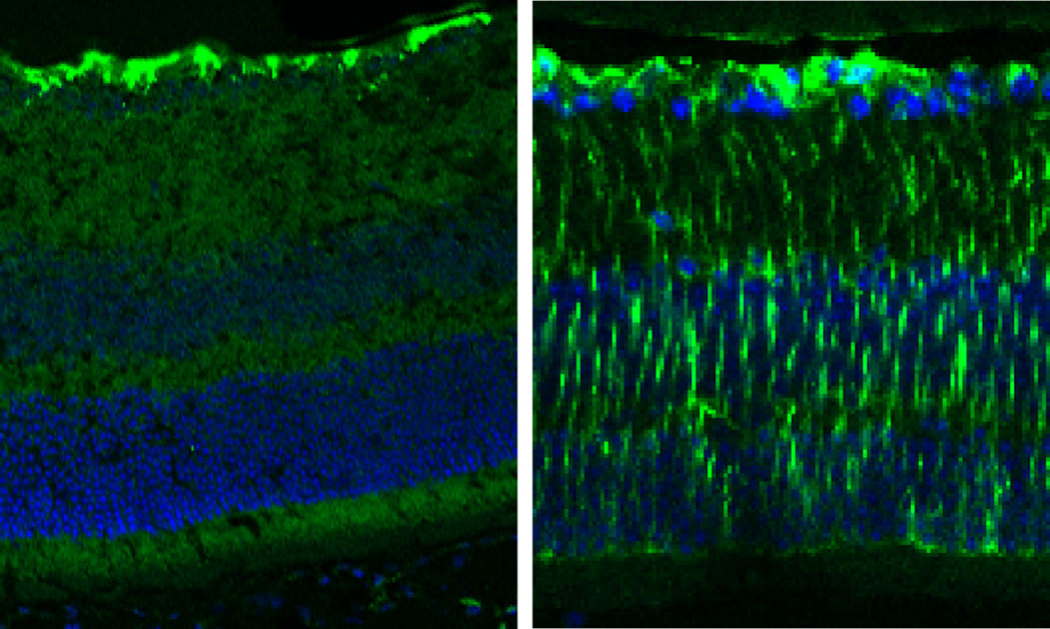
**Example of diseased retina undergoing a stress response – reactive gliosis.** Compared to the wild-type unstressed tissue (left) the mutant tissue shows an increase in GFAP staining. This is a valuable method to pinpoint when degeneration begins and allows us to target a specific time point before loss of light-sensitive photoreceptors can be seen, shown in the missense mutant (right).

**What's next for you?**

I hope to continue modelling human eye diseases to better understand the source of sight loss; in order to do this I am currently applying for funding.
